# International survey of treatment practices for atopic dermatitis in pregnant and breastfeeding women: Physician perspectives

**DOI:** 10.1111/ddg.15728

**Published:** 2025-06-08

**Authors:** Manuel P. Pereira, Katarina Stevanovic, Emek Kocatürk, Cathrin Meesch, Ingrid van Hofman, Prema S. Vaswani, Jonathan A. Bernstein, Dayanne Bruscky, Herberto J. Chong‐Neto, Chia‐Yu Chu, Roberta Fachini Jardim Criado, Luis Felipe Ensina, Ana M. Giménez‐Arnau, Kiran Godse, Maia Gotua, Stamatios Gregoriou, Kanokvalai Kulthanan, Charlotte G. Mortz, Natasa Teovska Mitrevska, Esen Özkaya, Prajwal Pudasaini, Mara Morelo Rocha Felix, Catalina Rincón Pérez, Claudio Alberto Salvador Parisi, Gonzalo N. Ramón, Efstratios Vakirlis, Zuotao Zhao, Lisa A. Beck, Marjolein de Bruin‐Weller, Michael Cork, Norito Katoh, Thomas Werfel, Margitta Worm, Andreas Wollenberg, Torsten Zuberbier

**Affiliations:** ^1^ Institute of Allergology Charité – Universitätsmedizin Berlin Corporate Member of Freie Universität Berlin and Humboldt‐Universität zu Berlin Berlin Germany; ^2^ Fraunhofer Institute for Translational Medicine and Pharmacology ITMP Immunology and Allergology Berlin Germany; ^3^ GA2LEN Global Allergy and Asthma Excellence Network Berlin Germany; ^4^ Bahcesehir University School of Medicine Department of Dermatology Istanbul Turkey; ^5^ University of Cincinnati College of Medicine Division of Rheumatology Allergy and Immunology Partner of Bernstein Allergy Group and Bernstein Clinical Research Center; ^6^ Allergy and Immunology Research Center Federal University of Pernambuco Recife Brazil; ^7^ Division of Allergy and Immunology‐Complexo Hospital de Clínicas‐Federal University of Paraná, Brazil; ^8^ Department of Dermatology National Taiwan University Hospital and National Taiwan University College of Medicine Taipei Taiwan; ^9^ Department of Dermatology Centro Universitário FMABC Alergoskin Alergia e Dermatologia – UCARE/ADCARE Center of Excellence – GA^2^LEN Santo André Brazil; ^10^ CPAlpha Clinical Research and Allergy Center Barueri Brazil; ^11^ Department of Dermatology Hospital del Mar Research Institute Universitat Pompeu Fabra Barcelona Spain; ^12^ D Y Patil University school of medicine Navi Mumbai India; ^13^ Center of Allergy and Immunology ADCARE Center Tbilisi Georgia; ^14^ David Tvildiani Medical University Tbilisi Georgia; ^15^ 1st Department of Dermatology and Venereology Andreas Sygros Hospital Faculty of Medicine National and Kapodistrian University of Athens Athens Greece; ^16^ Department of Dermatology Faculty of Medicine Siriraj Hospital Mahidol University Siriraj ADCARE Center Bangkok Thailand; ^17^ Department of Dermatology and Allergy Center Odense University Hospital Odense Denmark; ^18^ Department of Dermatology Remedika General Hospital Skopje North Macedonia; ^19^ Department of Dermatology and Venereology İstanbul Faculty of Medicine Istanbul University Istanbul Turkey; ^20^ Civil Service Hospital ADCARE Center Government of Nepal Kathmandu Nepal; ^21^ Alergolife ADCARE Center Rio de Janeiro Brazil; ^22^ GA^2^LEN Atopic Dermatitis Center of Reference and Excellence Medical Specialty Unit Secretaría de la Defensa Nacional Mexico City Mexico; ^23^ Pediatric and Adult Allergy Sections Italian Hospital of Buenos Aires Buenos Aires Argentina; ^24^ Instituto de Alergia e Inmunología del Sur Bahía Blanca Argentina; ^25^ 1st Department of Dermatology and Venereology Aristotle University of Thessaloniki Thessaloniki Greece; ^26^ Department of Dermatology and Venereology Peking University First Hospital Beijing Key Laboratory of Molecular Diagnosis on Dermatoses National Clinical Research Center for Skin and Immune Diseases NMPA Key Laboratory for Quality Control and Evaluation of Cosmetics Beijing China; ^27^ University of Rochester Medical Center Rochester New York USA; ^28^ Department of Dermatology and Allergology National Expertise Center for Atopic Dermatitis University Medical Center Utrecht Utrecht The Netherlands; ^29^ Sheffield Dermatology Research IICD University of Sheffield Sheffield UK; ^30^ North Campus Kyoto Prefectural University of Medicine Kyoto Japan; ^31^ Department of Dermatology and Allergy Hannover Medical School Hannover Germany; ^32^ Division of Allergy and Immunology Department of Dermatology and Allergology Charité – Universitätsmedizin Berlin Berlin Germany; ^33^ Department of Dermatology and Allergy Augsburg University Hospital Augsburg Germany; ^34^ Department of Dermatology and Allergy Ludwig Maximilian University of Munich Munich Germany; ^35^ Comprehensive Center for Inflammatory Medicine (CCIM) University Hospital Schleswig‐Holstein (UKSH) Lübeck Germany

**Keywords:** Atopic dermatitis, breastfeeding, pregnancy, survey, systemic treatment

## Abstract

**Background and Objectives:**

Systemic treatment of pregnant/breastfeeding atopic dermatitis (AD) patients is challenging due to limited safety data. We explored treatment practices with systemic agents, including the guideline‐recommended cyclosporine as the first systemic choice as well as emerging therapies, in this vulnerable population.

**Patients and Methods:**

The Global Allergy and Asthma Excellence Network (GA^2^LEN) ADCARE initiative collected data from physicians worldwide who treat pregnant women with AD. Physicians completed an electronic questionnaire on the use of systemic agents in pregnant/breastfeeding AD patients.

**Results:**

103 physicians from 32 countries completed the survey, primarily dermatologists (n = 48) or allergologists (n = 43). Antihistamines were the systemic drug most often considered to be used during pregnancy/breastfeeding (n = 73/81, 90.1%), with fewer physicians considering the use of systemic agents for the first trimester compared to later stages of pregnancy. For acute flares, systemic corticosteroids (n = 34/80, 42.5%) were preferred, followed by biologics and antihistamines (each n = 15/80, 18.8%). Although the guideline‐recommended cyclosporine is sometimes considered for AD during pregnancy (n = 38/81, 46.9%), it was rarely considered as the preferred drug by physicians (n = 1/80, 1.25%).

**Conclusions:**

Our study shows a misalignment between guideline recommendations and prescription patterns and highlights an unmet need for knowing and using the existing recommendations.

## INTRODUCTION

Atopic dermatitis (AD) is a chronic, highly pruritic, systemic inflammatory skin disease.^1^, ^2^ It is associated with many comorbidities and significantly impacts the patient's quality of life.^3^ Patients are not only affected by the social stigma of a visible skin condition, but also by intense pruritus, which leads to skin trauma and significant sleep disturbances. These factors contribute to psychological stress, which in turn exacerbates itching and triggers disease flares, creating a vicious cycle.^1^, ^4^ The treatment of AD is multifaceted and includes measures to strengthen the skin barrier, topical anti‐inflammatory and antipruritic therapies, antibacterial strategies, and systemic treatment. Systemic options comprise modern biologics (e.g., dupilumab, tralokinumab, lebrikizumab), Janus kinase (JAK) inhibitors (e.g., abrocitinib, baricitinib, upadacitinib), and conventional immunosuppressants (e.g., cyclosporine, methotrexate, azathioprine). These approaches aim to achieve disease control and prevent the development of comorbidities.^1^, ^5^, ^6^

Atopic dermatitis is the most common skin disease occurring during pregnancy, accounting for up to 50% of all pregnancy‐related dermatoses.^7^, ^8^ Changes in hormone levels influence cytokine balance and can lead to *de novo* manifestation of eczematous lesions, referred to as atopic eruption of pregnancy.^7^ Existing AD prior to pregnancy is also reported to worsen during pregnancy in about 50% of AD patients.^9^

Treatment options for pregnant or lactating AD patients are limited due to teratogenic effects or to lack of safety data in pregnancy cohorts.^10^ Surveys conducted in the UK have reported that over 70% of pregnant individuals with AD deliberately avoid medication due to concerns about potential harm to the fetus.^11^ Treatment with antihistamines is still reported in these patients, despite the fact that atopic dermatitis‐associated pruritus is largely histamine‐independent and antihistamines have little effect on underlying inflammation.^7^ In contrast, both the *European Task Force on Atopic Dermatitis* (ETFAD) and the current *European Guidelines for Atopic Dermatitis* (EuroGuiDerm guidelines) recommend cyclosporine as first line treatment during pregnancy or breastfeeding in severe cases of AD.^5^, ^6^, ^10^ However, with emerging therapies, a document titled “Safety of dermatologic medications in pregnancy and lactation” has been initiated with periodic updates to review safety profiles of novel therapeutics for pregnant or lactating women.^12^ Moreover, pregnancy exposure registries including patients with atopic dermatitis treated with dupilumab, tralokinumab, abrocitinib, and ruxolitinib cream are currently ongoing (see list at the FDA Pregnancy Exposure Registries). Data of treatment characteristics and pregnancy outcomes in women with AD was collected in Denmark and in the United states,^13^, ^14^ while recent population‐based studies investigated outcomes of pregnancy in AD patients without analyzing what treatments were performed.^15^, ^16^ It remains unclear which systemic treatments are favored in clinical settings outside of Europe or the US. The consensus on systemic treatment during pregnancy has already been established.^17^ However, this study aims to understand the unmet need in medical education by exploring, at an international level, the treatment practices of physicians who routinely treat AD patients.

The ADCARE network consists of specialized centers for the treatment of AD. It is affiliated with the *Global Allergy and Asthma Excellence Network* (GA^2^LEN), the largest multidisciplinary network of research centers and clinical care in allergy and asthma, conducting research and educational activities.

This survey investigated the systemic treatment patterns of physicians treating pregnant or lactating women with AD and the question of whether these approaches align with local treatment guidelines^5^, ^6^, ^18^, ^19^, ^20^, ^21^, ^22^ and the latest safety knowledge. These findings are intended to initiate the establishment of a global consensus on optimal AD treatment approaches for pregnant and lactating patients.

## PATIENTS AND METHODS

### Study Design

Physicians within the ADCARE network who regularly treat AD patients were invited to participate in a survey in the English language about systemic treatments for AD used in pregnancy or breastfeeding, as supplied in online supplementary Figure S1. Physician participation was voluntary after being informed about the aims and nature of the study. There were no risks or disadvantages for the participating physicians including the choice to refuse participation, and the participating physician could withdraw from the project at any time for any reason. The participating physicians were offered the possibility to invite non‐ADCARE physicians to take part in the survey. Institutional review board approval was waived because no patients were involved in this study.

## STUDY OUTCOMES

The survey questionnaire consisted of six questions, five of which are reported in this article: *(1)* medical specialization; *(2)* number of treated pregnant AD patients in the previous three years; *(3)* systemic treatment options for AD considered during each trimester of pregnancy or breastfeeding (including systemic corticosteroids for acute use, systemic corticosteroids for long‐term use, systemic immunosuppressants [azathioprine, cyclosporine, methotrexate, mycophenolate mofetil, other], biologics [dupilumab, tralokinumab, other], JAK inhibitors [abrocitinib, baricitinib, upadacitinib, other], antihistamines [sedating, non‐sedating], other medication); *(4)* systemic treatment of choice; *(5)* complications regarding the outcome of pregnancy (prematurity, preterm delivery, malformations, fetal loss, other). Topical agents and phototherapy were not covered in this survey.

### Data collection and analysis

The data were anonymized and collected in a central database from 10 June 2023 to 23 November 2023. Missing data was handled using complete case analysis. Statistical analyses were performed using IBM SPSS Statistics for Windows, version 27 (Armonk, NY, USA). Data are shown as number of cases/total number of assessments (percentage of cases).

## RESULTS

### Respondents

**FIGURE 1:**
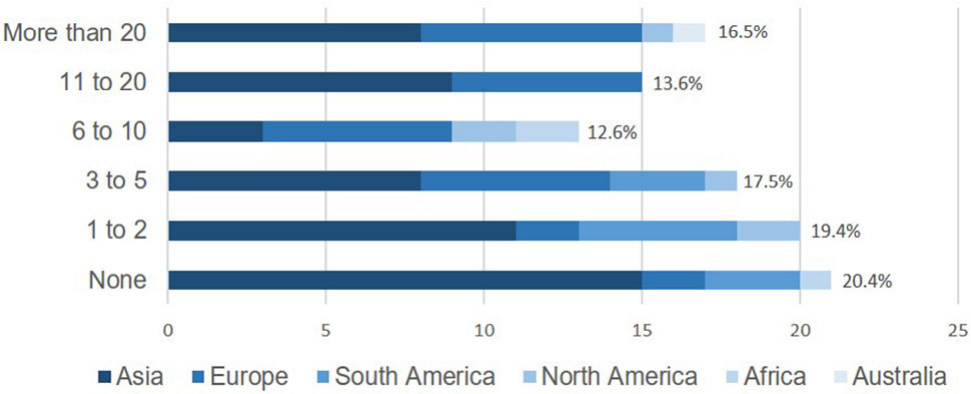
Number of pregnant patients with atopic dermatitis treated in the previous three years. X‐axis: number of respondents.

A total of 103 physicians from 32 countries across six continents completed the survey. Most respondents were from Asia (n = 54) and Europe (n = 28), while Thailand was the most represented country (n = 25), followed by India (n = 8), Brazil (n = 8), Portugal (n = 7) and Germany (n = 7). Most physicians were either dermatologists (n = 48) or allergologists (n = 43), with a wide range of pregnant AD patients treated over the past three years (Figure 1). A subset of respondents (n = 81) completed the whole questionnaire. The distribution of these responders by continent, country, specialty and experience in treating pregnant patients with AD is shown in Table 1.

**TABLE 1:** Distribution of responders by continent, country, specialty and experience in treating pregnant patients with atopic dermatitis. Data from respondents completing the whole questionnaire is shown.

Continent	Number of respondents	Country	Specialty	Treated patients in the previous 3 years
Asia	n = 39	Thailand: n = 17, India: n = 5, Vietnam: n = 4, Nepal: n = 3, China: n = 2, Israel: n = 2, Japan: n = 1, Malaysia: n = 1, Philippines: n = 1, Taiwan: n = 1, Turkey^*^: n = 1, United Arab Emirates: n = 1	Dermatology: n = 17 Allergology: n = 15 Internal medicine: n = 1 GP: n = 6	> 20: n = 5 11–20: n = 8 6–10: n = 3 3–5: n = 6 1–2: n = 9 None: n = 8
Europe	n = 24	Portugal: n = 6, Germany: n = 5, Greece: n = 2, Italy: n = 2, Russia: n = 2, Turkey^*^: n = 2, Austria: n = 1, Denmark: n = 1, Macedonia: n = 1, Spain: n = 1, Switzerland: n = 1	Dermatology: n = 15 Allergology: n = 9	> 20: n = 6 11–20: n = 4 6–10: n = 6 3–5: n = 5 1–2: n = 2 None: n = 1
South America	n = 10	Brazil: n = 8, Argentina: n = 2	Dermatology: n = 1 Allergology: n = 9	> 20: n = 0 11–20: n = 0 6–10: n = 0 3–5: n = 3 1–2: n = 4 None: n = 3
North America	n = 5	United States of America: n = 3, Canada: n = 1, Mexico: n = 1	Dermatology: n = 4 Allergology: n = 1	> 20: n = 1 11–20: n = 0 6–10: n = 2 3–5: n = 1 1–2: n = 1 None: n = 0
Africa	n = 2	Madagascar: n = 1, South Africa: n = 1	Dermatology: n = 1 Allergology: n = 1	> 20: n = 0 11–20: n = 0 6–10: n = 2 3–5: n = 0 1–2: n = 0 None: n = 0
Australia	n = 1	Australia: n = 1	Allergology: n = 1	> 20: n = 1 11–20: n = 0 6–10: n = 0 3–5: n = 0 1–2: n = 0 None: n = 0

*Abbr*.: GP, general practitioner

* Turkey is either considered being part of Asia or Europe according to respondents’ assessment.

### Systemic treatments

**FIGURE 2:**
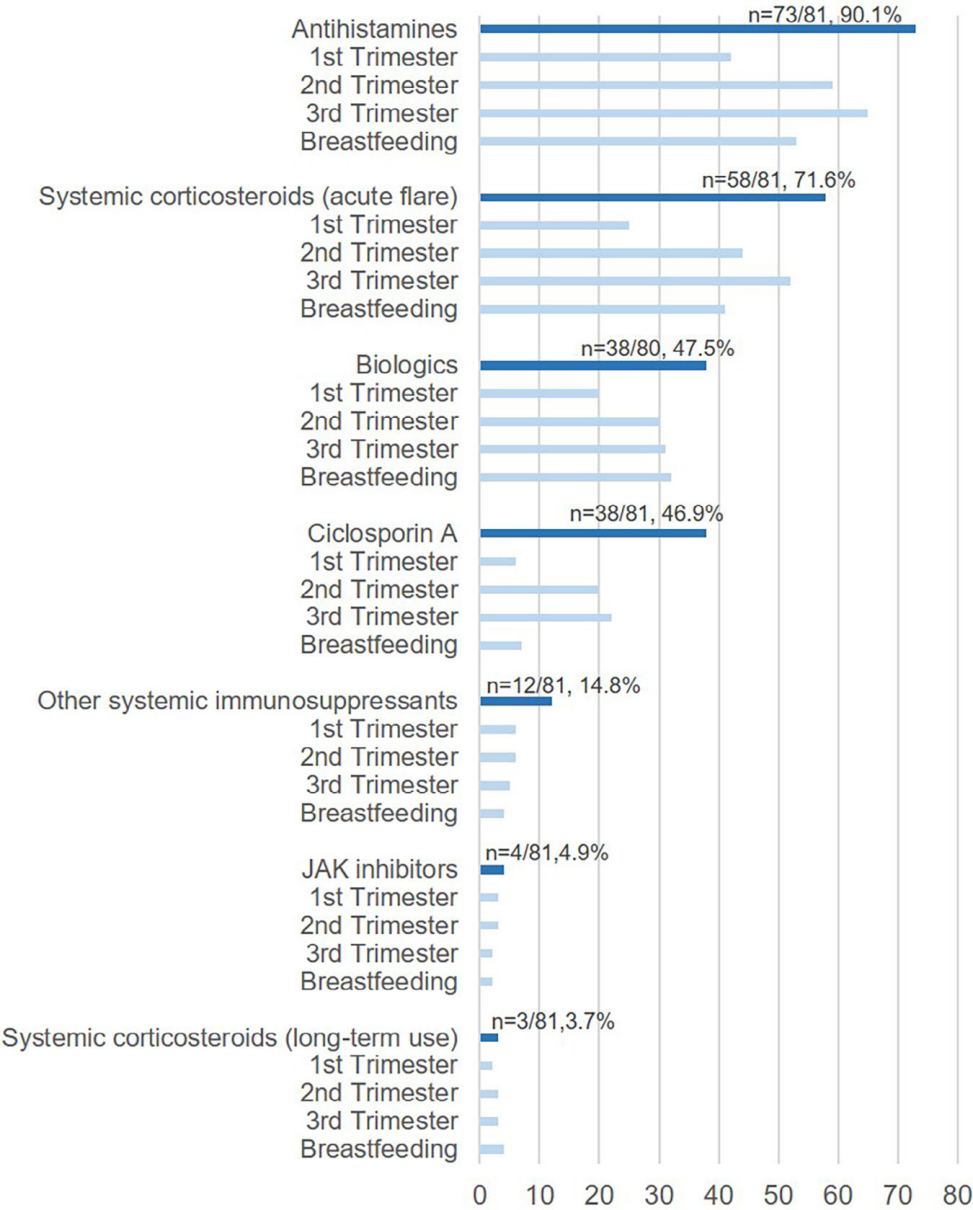
Systemic treatments used during pregnancy/breastfeeding. The number of physicians considering the use of antihistamines, systemic corticosteroids for acute flares, biologics, systemic immunosuppressive drugs (including cyclosporine), Janus kinase (JAK) inhibitors and systemic corticosteroids as a long‐term treatment to treat atopic dermatitis during pregnancy and breastfeeding is shown. X‐axis: number of respondents.

Antihistamines were generally considered a therapy option throughout pregnancy and breastfeeding (n = 73/81, 90.1%), although fewer respondents considered them suitable in the first trimester of pregnancy (Figure 2). Mostly non‐sedating antihistamines (n = 56) were preferred, while sedating (n = 19) antihistamines were chosen less often.

Short‐term use of systemic corticosteroids for acute flares was also regarded as feasible by most physicians (n = 58/81, 71.6%), but only 25 physicians considered using them in the first trimester (Figure 2).

Although not licensed for use in pregnancy or lactation, biologics were a possible therapeutic option for a sizable number of physicians (n = 38/80, 47.5%), with dupilumab (n = 42) being the biologic of choice, followed by tralokinumab (n = 9) and other biologics (n = 2). Again, fewer physicians regarded biologics as a treatment option for the first trimester compared to later trimesters or during lactation.

A minority of respondents regarded systemic immunosuppressants as a therapy option during pregnancy or lactation (n = 19/81, 23.5%). Cyclosporine (n = 38) was most often considered, followed by azathioprine (n = 11). Of note, immunosuppressants or immunomodulators with known teratogenic effects were also mentioned by a few respondents: methotrexate (n = 5), JAK inhibitors (n = 4), and mycophenolate mofetil (n = 3).

Long‐term corticosteroid use was rarely considered for the treatment of AD during pregnancy or breastfeeding (n = 3/81, 3.7%), while n = 27/80 (33.8%) did not use any systemic agent during pregnancy or breastfeeding.

### Preferred systemic treatments

**FIGURE 3:**
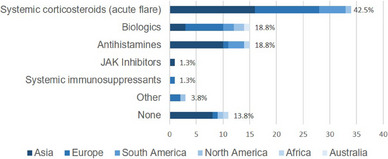
Preferred systemic treatment for atopic dermatitis during pregnancy/breastfeeding. The number of physicians with preference for the use of systemic corticosteroids for acute flares, biologics, antihistamines, Janus kinase (JAK) inhibitors, systemic immunosuppressive drugs, and other systemic agents to treat atopic dermatitis during pregnancy and breastfeeding is shown. X‐axis: number of respondents.

Eighty physicians answered the question regarding their preferred systemic treatment in AD during pregnancy or breastfeeding (Figure 3). Systemic corticosteroids for acute flares were the most frequent answer (n = 34, 42.5%), followed by biologics (n = 15, 18.8%) and antihistamines (n = 15, 18.8%). Cyclosporine and baricitinib were only chosen by one respondent each as the systemic drug of choice.

Dupilumab was most frequently mentioned as the biologic of choice (n = 13), while tralokinumab was mentioned by one respondent (n = 1), and one respondent's data was missing (n = 1). Non‐sedating antihistamines (n = 8) were more often reported as the antihistamine of choice in comparison with sedating antihistamines (n = 3).

Eleven (13.8%) respondents revealed that they prefer not to use any systemic agent to treat AD during pregnancy or breastfeeding.

### Complications

Only six respondents reported complications during pregnancy associated with the use of systemic therapies. Prematurity and preterm delivery were each reported by two physicians, while malformations, fetal loss, and unspecified complications were each reported once.

## DISCUSSION

The clinical management of AD patients during pregnancy and breastfeeding remains challenging, particularly when systemic agents are needed. Our international study revealed systemic treatment trends by physicians from across the globe. Primarily, it was noted that some physicians show reluctance in providing systemic drugs during the first trimester since fetal development is very critical at this stage of pregnancy. Non‐sedating antihistamines are the drug most perceived by physicians as safe for use during pregnancy or breastfeeding, and their wide use reflects the overwhelming safety data available for this drug class.^23^ However, older‐generation antihistamines have an unknown effect on the fetus and should not be prescribed.^24^ Moreover, antihistamines show limited anti‐inflammatory and antipruritic effects, since non‐histaminergic nerve fibers are responsible for conducting atopic itch,^25^, ^26^, ^27^ and thus their usefulness in AD treatment is limited, both as monotherapy or add‐on therapy.^28^ Accordingly, antihistamines are not recommended for the treatment of AD by the ETFAD and EuroGuiDerm guideline group.^5^, ^6^, ^17^

Systemic corticosteroids use for the treatment of acute flares is favored by most physicians, however, there are concerns about suppression of the hypothalamus‐pituitary‐adrenal axis in newborns induced by long‐term corticosteroid use.^17^, ^29^

Biologics, especially dupilumab, also seem to enjoy wide acceptance with a preference for its use in later stages of pregnancy, thus avoiding critical periods of fetal development. However, a retrospective analysis of exposure prior to or during the first 6 weeks of pregnancy in women with AD revealed that there was no significant drug‐associated risk for adverse pregnancy, congenital, neonatal, or post‐partum outcomes during the first trimester.^30^ In the treatment of other indications such as asthma, it is common to start biologics in patients who responded to them before pregnancy.^31^ Importantly, as biologics used in AD target Th2 inflammation, which is beneficial for sustaining pregnancy, they may promote a shift towards Th1 inflammation. Since Th1 cytokines may contribute to complications such as pre‐eclampsia or pre‐term birth and harm the fetus, caution should be taken when choosing a treatment option targeting Th2 inflammation. Nevertheless, currently available data do not suggest that Th2‐blocking biologics would influence fetal or maternal outcomes, but high‐quality controlled studies are needed for more robust evidence.^32^, ^33^, ^34^ Compared to Asia, in Europe a higher proportion of physicians tend to favor the use of biologics, indicating possible differences in attitudes towards systemic treatment during pregnancy and breastfeeding across cultures. In addition, varying access to modern systemic agents and different reimbursement policies by health insurance providers in the respective regions could play a role.

On the other hand, most broad‐acting systemic immunosuppressive drugs and JAK inhibitors are not favored by most physicians who responded to this questionnaire, as some of these substances are still perceived to carry potential risks for both the mother and the fetus. Importantly methotrexate,^35^ mycophenolate mofetil,^36^ and JAK inhibitors^37^ are teratogenic and contraindicated in pregnancy by label, also as described in the American AD guidelines.^19^ On the other hand, for cyclosporine there is robust evidence of its safety in pregnancy and breastfeeding^38^, ^39^ and it is recommended by both the ETFAD and the current European guidelines for AD.^5^, ^6^, ^17^ A panel of Canadian experts provided in 2023 literature‐review‐based recommendations for systemic treatment of AD, which stated that for pregnant or lactating women needing systemic treatment, cyclosporine has the most evidence supporting its use, while methotrexate, mycophenolate mofetil, and JAK inhibitors are contraindicated.^19^, ^40^ Cyclosporine is also confidently used for treatment of other indications, such as in kidney transplantation and irritable bowel disease during pregnancy.^41^, ^42^, ^43^

Remarkably, a substantial proportion of physicians revealed not using any systemic agents to treat AD during pregnancy or breastfeeding, underscoring the challenge physicians face in managing AD symptoms, while taking into account potential risks for the mother and child.

Additionally, complications including prematurity, malformations and fetal loss were reported by a few physicians. Notably, with our study design, none of the reported complications could be attributed to a specific systemic drug and thus these results should be regarded with caution. Other limitations of the study include having a relatively low number of participants and the limited global coverage, with little data available in particular for Australia (n = 1), Africa (n = 2), and North America (n = 5), as described in Table 1.

Our findings indicate that current recommendations for the systemic treatment of AD during pregnancy and breastfeeding are not consistently followed by local physicians treating this patient population. The discrepancies observed in our survey responses are likely attributable to varying levels of expertise among respondents, regional differences in the treatment of pregnant and breastfeeding patients, and concerns among physicians and patients about potential side effects and complications, such as renal failure associated with cyclosporine therapy. Consequently, peer education efforts, such as ADCARE training courses,^44^, ^45^ should be implemented more frequently and comprehensively to achieve better universal care in this vulnerable patient population with moderate to severe AD. Post‐event analysis has shown a 43% increase in test results compared to the level of knowledge prior to the event.^44^ For effective guidance, guidelines should take regional particularities into account, including access to modern medication and reimbursement policies by insurance providers. We have previously discussed the complexity of AD and the benefits of involving various stakeholders in treatment and patient education,^46^ and have therefore developed the AD Integrated Care Pathways as an additional resource for both patients and physicians.^1^ With the current study, ADCARE has gained insights into existing knowledge gaps among physicians and has identified topics that should be addressed more thoroughly in future training events.

## CONCLUSIONS

Our study revealed a discrepancy between guideline recommendations and physician prescription patterns for systemic drugs used to treat pregnant and lactating women with AD. Educational programs on treatment updates for specific patient groups, along with long‐term registries collecting data on the use, efficacy, and safety of systemic agents during pregnancy and breastfeeding, are needed to inform future guidelines and support treating physicians.

## FUNDING

This study was funded by Almirall. The sponsor provided financial support for the study but had no role in the design of the study protocol, data collection, analysis, interpretation, or the decision to publish this manuscript. The authors retained full control over all aspects of the study and manuscript preparation.

## CONFLICT OF INTEREST STATEMENT

M.P.P. has received research funding from Almirall; is an investigator for Allakos, Celldex Therapeutics, Incyte, Sanofi, and Trevi Therapeutics; and has received consulting fees, speaker honoraria, and/or travel fees from AbbVie, Beiersdorf, Celltrion, Eli Lilly, GA^2^LEN, Galderma, Menlo Therapeutics, Novartis, P.G. Unna Academy, Sanofi, StreamedUP, and Trevi Therapeutics. E.K. has acted as a speaker and advisor for Novartis, Menarini, and Pfizer. M.D.B. has been a consultant, advisory board member, and/or speaker for AbbVie, Almirall, Amgen, Aslan, Eli Lilly, Galderma, Leo Pharma, Pfizer, Regeneron, and Sanofi‐Genzyme. N.K. has received honoraria as a speaker/consultant for Sanofi, Maruho, AbbVie, Eli Lilly Japan, Taiho Pharmaceutical, Pfizer, Mitsubishi Tanabe Pharma, Janssen Pharma, Kyowa Kirin, Celgene Japan, and Otsuka Pharmaceutical, and has received investigator‐initiated grants from Mitsubishi Tanabe Pharma, Torii Pharmaceutical, Maruho, Sun Pharma, Boehringer Ingelheim Japan, Eisai, and Leo Pharma. M.W. reports support for consultancies, lectures, and other scientific activities from ALK‐Abelló Arzneimittel GmbH, Almirall, AbbVie, Eli Lilly, Mylan Germany GmbH, Bencard Allergie GmbH, Novartis AG, Biotest AG, Sanofi‐Aventis Deutschland GmbH, HAL Allergie GmbH, DBV Technologies S.A., Aimmune Therapeutics UK Limited, Regeneron Pharmaceuticals Inc., and Stallergenes GmbH. T.W. has received institutional research grants from Almirall, Beiersdorf, LEO Pharma, and Novartis; has performed consultancies for AbbVie, Almirall, Galderma, LEO, Lilly, Novartis, Pfizer, and Sanofi‐Regeneron; has lectured at educational events sponsored by AbbVie, Almirall, Galderma, LEO Pharma, Lilly, Pfizer, Sanofi, and Novartis; and is involved in clinical trials conducted with various pharmaceutical companies developing treatments for atopic dermatitis. A.W. has served as an advisor or paid speaker for, or participated in clinical trials (with honoraria paid to the institution) sponsored by AbbVie, Aileens, Almirall, Amgen, Beiersdorf, Bioderma, Bioproject, Boehringer Ingelheim, Bristol Myers Squibb, Celgene, Chugai, DKSH, Eli Lilly, Galapagos, Galderma, Glenmark, GSK, Hans Karrer, Hexal, Janssen‐Cilag, Kyowa Kirin, Leo Pharma, L'Oréal, Maruho, MedImmune, MSD, Mylan, Novartis, Pfizer, Pierre Fabre, Regeneron, Sandoz, Santen, Sanofi‐Aventis, and UCB. T.Z. reports honoraria for lectures from Amgen, AstraZeneca, AbbVie, ALK‐Abelló, Almirall, Astellas, Bayer HealthCare, Bencard, Berlin Chemie, FAES Farma, HAL Allergie GmbH, Henkel, Kryolan, Leti, L'Oréal, Meda, Menarini, Merck Sharp & Dohme, Novartis, Nuocor, Pfizer, Sanofi, Stallergenes, Takeda, Teva, UCB, and Uriach. Fees for industry consulting were received from Abivax, Almirall, Bluprint, Celldex, Celltrion, Novartis, and Sanofi. In addition, he declares non‐paid organizational affiliations: committee member, “Allergic Rhinitis and its Impact on Asthma” (ARIA); member of the board, German Society for Allergy and Clinical Immunology (DGAKI); head, European Centre for Allergy Research Foundation (ECARF); president, Global Allergy and Asthma Excellence Network (GA^2^LEN); and member, Committee on Allergy Diagnosis and Molecular Allergology, World Allergy Organization (WAO). C.Y.C. has received research funding from Sanofi; is an investigator for AbbVie, Amgen, Dermira, Janssen, Eli Lilly, Novartis, Oneness Biotech, Pfizer, Regeneron Pharmaceuticals Inc., Roche, and Sanofi; and has received consulting fees, speaker honoraria, and/or travel fees from AbbVie, Amgen, Eli Lilly, Janssen, Novartis, Pfizer, Roche, Sanofi, and Viatris. A.G.A. is or recently was a speaker and/or advisor for, and/or has received research funding from, Almirall, Amgen, AstraZeneca, Avène, Bluprint, Celldex, Escient Pharmaceuticals, Genentech, GSK, Harmonic Bio, Instituto Carlos III–FEDER, Jaspers, Leo Pharma, Menarini, Mitsubishi Tanabe Pharma, Novartis, Sanofi–Regeneron, Septerna, Servier, Thermo Fisher Scientific, Uriach Pharma, and Noucor. L.F.E. received consulting fees from Sanofi, speaker honoraria from Sanofi, Novartis, and AbbVie, and participates in clinical trials from Novartis and Amgen. K.K. received speaker honoraria from Pfizer, Zuellig Pharma, and Sanofi. E.Ö. has acted on advisory boards for Pfizer and Sanofi and has received speaker honoraria from both companies. C.R.P. is an investigator for AbbVie and has received consulting fees, speaker honoraria, and/or travel fees from AbbVie, Eli Lilly, Leo Pharma, and Janssen‐Cilag. K.S., C.M., I.V.H., P.V., L.B., D.B., H.C., K.G., M.G., S.G., C.M., N.T.M., P.P., M.M.R.F., C.A.S.P., G.R., and E.V. declare no conflicts of interest.

## Supporting information



Supplementary information
