# Supplementary material for: International survey of treatment practices for atopic dermatitis in pregnant and breastfeeding women: Physician perspectives
Source: J Dtsch Dermatol Ges. 2025 Jun 8;23(9):1116–24. doi: 10.1111/ddg.15728 (PMC12435135; doi:10.1111/ddg.15728)
Supplement: Supplementary file 1 — Supplementary information [file DDG-23-1116-s001.docx]

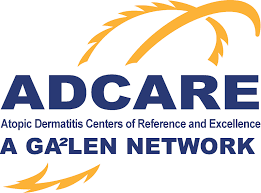


Physician:

Center:

Systemic Treatment of Atopic Dermatitis in Pregnancy: A Physician Questionnaire

Dear Colleague

The treatment of atopic dermatitis during pregnancy remains a challenge, especially when systemic agents are required. The ADCARE (Atopic Dermatitis Centres of Reference and Excellence) Network aims to perform a study that determines the systemic treatment of choice for atopic dermatitis during pregnancy. For this purpose, we would like you to complete a short survey (6 questions) regarding your experience treating pregnant patients with atopic dermatitis, your systemic treatment of choice during pregnancy and the side-effects you experienced using the chosen medication. Please do not leave any questions unanswered and note that some questions may have more than one answer.

Thank you for your participation.

**PHYSICIAN QUESTIONNAIRE**

1. What is your medical specialization?
   - Allergologist
   - Dermatologist
   - Gynecologist
   - Specialist in internal medicine
   - General practitioner
   - Other. Please specify:
2. How many pregnant patients with atopic dermatitis have you treated in the last three years?
   - None
   - 1-2
   - 3-5
   - 6-10
   - 11-20
   - >20
3. Which of the following systemic treatment options for atopic dermatitis would you consider safe for a pregnant patient according to the trimesters of pregnancy, or during breastfeeding? You can select several options.
   - I do not use any systemic treatments during the following trimesters of pregnancy or during breastfeeding:
     - First trimester
     - Second trimester
     - Third trimester
     - During breastfeeding
   - Systemic corticosteroids for acute flare use:
     - First trimester
     - Second trimester
     - Third trimester
     - During breastfeeding
   - Systemic corticosteroids for long term use:
     - First trimester
     - Second trimester
     - Third trimester
     - During breastfeeding
   - Biologics:
     - Dupilumab
     - Tralokinumab
     - Other:
     - First trimester
     - Second trimester
     - Third trimester
     - During breastfeeding
   - JAK Inhibitors:
     - Abrocitinib
     - Baricitinib
     - Upadacitinib
     - Other:
       - First trimester
       - Second trimester
       - Third trimester
       - During breastfeeding
   - Systemic immunosuppressants:
     - Azathioprine
     - Cyclosporine A
     - Methotrexate
     - Mycophenolate Mofetil
     - Other:
       - First trimester
       - Second trimester
       - Third trimester
       - During breastfeeding
   - Antihistamines:
     - Non-sedating antihistamines (Please specify the drug name: )
     - Sedating antihistamines (Please specify the drug name: )
       - First trimester
       - Second trimester
       - Third trimester
       - During breastfeeding
   - Other:
     - First trimester
     - Second trimester
     - Third trimester
     - During breastfeeding
4. Which of the following options is your preferred systemic treatment of choice to treat atopic dermatitis according to the trimesters of pregnancy or during breastfeeding? Please select only one option regarding the treatment of choice; multiple options are possible regarding the trimesters of pregnancy/breastfeeding you use your treatment of choice.
   - I do not use any systemic treatments during pregnancy/breastfeeding.
     - First trimester
     - Second trimester
     - Third trimester
     - During breastfeeding
   - Systemic corticosteroids for acute flare use:
     - First trimester
     - Second trimester
     - Third trimester
     - During breastfeeding
   - Systemic corticosteroids for long term use:
     - First trimester
     - Second trimester
     - Third trimester
     - During breastfeeding
   - Biologics:
     - Dupilumab
     - Tralokinumab
     - Other:
       - First trimester
       - Second trimester
       - Third trimester
       - During breastfeeding
   - JAK Inhibitors:
     - Abrocitinib
     - Baricitinib
     - Upadacitinib
     - Other:
       - First trimester
       - Second trimester
       - Third trimester
       - During breastfeeding
   - Systemic immunosuppressants:
     - Azathioprine
     - Cyclosporine A
     - Methotrexate
     - Mycophenolate Mofetil
     - Other:
       - First trimester
       - Second trimester
       - Third trimester
       - During breastfeeding
   - Antihistamines
     - Non-sedating antihistamines (Please specify the drug name: )
     - Sedating antihistamines (Please specify the drug name: )
       - First trimester
       - Second trimester
       - Third trimester
       - During breastfeeding
   - Other:
     - First trimester
     - Second trimester
     - Third trimester
     - During breastfeeding
5. Which of the following side-effects have you observed with your systemic treatment of choice in atopic dermatitis during pregnancy (answer to Q4)? You can select several options.
   - Cardiovascular side-effects (e.g. high-blood pressure, arrhythmia, palpitations)
   - Systemic infections (e.g. common flu, bronchitis, pneumonia)
   - Neurological side-effects (e.g. headache, dizziness, tiredness, numbness, tingling)
   - Gastroenterological side-effects (e.g. nausea, vomiting, diarrhea, stomach ulcer)
   - Hepatic side-effects (e.g. high level of liver enzymes, hepatitis, pancreatitis, cholestasis)
   - Nephrological side-effects (e.g. renal insufficiency)
   - Endocrinological side-effects (e.g. dyslipidemia, diabetes including gestational diabetes, enhanced hair growth, weight gain/loss)
   - Hematological side-effects (e.g. anemia, changes in complete blood count)
   - Dermatological side-effects (e.g. rash, itch, acne, flush, allergic reactions, skin infections, local reactions)
   - Ophthalmological side-effects (e.g. conjunctivitis, keratitis, blurred vision)
   - Thromboembolic side-effects (e.g. deep vein thrombosis, pulmonary embolism)
   - Musculoskeletal side-effects (e.g. muscle pain, cramps, pain in the joints, increased levels of creatine kinase)
   - Psychiatric side-effects (e.g. depression, anxiety, suicidal ideation)
   - Other:
   - Please specify the observed side-effects:
6. Have you ever observed any of the following complications regarding the outcome of pregnancy associated with the use of above-mentioned systemic treatments? You can select several options.
   - There were no complications.
   - Prematurity
   - Preterm delivery
   - Malformations
   - Fetal loss
   - Other. Please specify:

Any additional comments:
